# Overlapping Structures Detection in Protein-Protein Interaction Networks Using Community Detection Algorithm Based on Neighbor Clustering Coefficient

**DOI:** 10.3389/fgene.2021.689515

**Published:** 2021-06-23

**Authors:** Yan Wang, Qiong Chen, Lili Yang, Sen Yang, Kai He, Xuping Xie

**Affiliations:** ^1^Key Laboratory of Symbol Computation and Knowledge Engineering of Ministry of Education, College of Computer Science and Technology, Jilin University, Changchun, China; ^2^School of Artificial Intelligence, Jilin University, Changchun, China; ^3^Department of Obstetrics, The First Hospital of Jilin University, Changchun, China

**Keywords:** protein-protein interaction network, overlapping structure, clustering coefficient, community detection, central edge

## Abstract

With the rapid development of bioinformatics, researchers have applied community detection algorithms to detect functional modules in protein-protein interaction (PPI) networks that can predict the function of unknown proteins at the molecular level and further reveal the regularity of cell activity. Clusters in a PPI network may overlap where a protein is involved in multiple functional modules. To identify overlapping structures in protein functional modules, this paper proposes a novel overlapping community detection algorithm based on the neighboring local clustering coefficient (NLC). The contributions of the NLC algorithm are threefold: (i) Combine the edge-based community detection method with local expansion in seed selection and the local clustering coefficient of neighboring nodes to improve the accuracy of seed selection; (ii) A method of measuring the distance between edges is improved to make the result of community division more accurate; (iii) A community optimization strategy for the excessive overlapping nodes makes the overlapping structure more reasonable. The experimental results on standard networks, Lancichinetti-Fortunato-Radicchi (LFR) benchmark networks and PPI networks show that the NLC algorithm can improve the Extended modularity (EQ) value and Normalized Mutual Information (NMI) value of the community division, which verifies that the algorithm can not only detect reasonable communities but also identify overlapping structures in networks.

## Introduction

Due to the rapid development of experimental and computing technology, a large number of PPI networks have been mined (Chen et al., 2020). Previous studies have reported that a PPI network can be constructed as a scale-free complex network and satisfies small-world property and high degree of clustering (Ji et al., 2012). Biological functions are performed by many functionally related proteins. Such clustering proteins are called functional module. A module represents a group of proteins taking part in specific, separable functions such as protein complexes, metabolic pathways or signal transduction systems (Vella et al., 2018). Lots of overlapping structures are shared by the functional modules in PPI networks, indicating some proteins play indispensable roles in different biological processes (Gu et al., 2019). Research on detecting protein functional modules has become one of the most important topics in both life science and computing science since the completion of the Human Genome Project (Ying and Lin, 2020). Detecting overlapping structures in functional modules have good application prospects in protein biological function, disease-causing gene, and drug target prediction.

In recent years, many researchers have designed a large number of algorithms that use the computational methodology to identify overlapping structures in modules. Among myriads of such efforts, network clustering is one of the most popular approaches for analyzing the topological and functional properties of PPI networks (Bhowmick and Seah, 2015). For example, the cluster percolation method (CPM) was the first method to discover overlapping communities. Its main idea was to determine k-connected subgraphs in the network and regard k-connected subgraphs as communities (Palla et al., 2005). By setting different *k* values, communities with different sizes can be obtained. The clustered communities will overlap, but the division result depends on the value of parameter *k*. Another common strategy used for community detection was based on edge division. This idea was initially used by Ahn et al., who proposed the classic link clustering community detection algorithm (LC) (Ahn et al., 2010). The LC algorithm first used the classical Jacard distance formula to quantify the distance between edges. The hierarchical clustering method was used to obtain the hierarchical structure of the community, and then the hierarchical structure was cut using the division function of density. Although there were many overlapping structures in the LC algorithm, the division result was quite different from the real community structure. In 2016, based on the density peak clustering algorithm, Huang et al. proposed a novel node distance measurement based on node similarity and the shortest distance between nodes, which could measure the global distance in the network, and applied the density peak clustering algorithm to the community of the detection network structure (Huang et al., 2016). In 2017, Qi et al. (2017). proposed an overlapping community detection algorithm based on the selection of seed nodes (CNS). The two main processes of the CNS algorithm were the selection of the central node and the clustering process. In 2018, Zhang et al. proposed an overlapping community discovery algorithm based on central edge selection (CES) (Zhang et al., 2018). The algorithm introduced the theory of community magnetic interference (CMI), which reduced the probability of the neighboring nodes becoming a central node and made the target central node reliable. Nevertheless, the division result was not sufficiently accurate.

Though the detection of functional modules in PPI networks has aroused widespread attention over the past few years, how to design correct and effective functional module detection methods is still a challenging and important scientific problem in computational biology (Mao and Liu, 2020). One of the main obstacles in community discovery is the accuracy of the division results. To improve the accuracy of community division, this paper proposes an overlapping community detection algorithm based on the neighbor local clustering coefficient (NLC) to select the central edge. The NLC algorithm introduces the clustering coefficient to improve the selection of seeds and optimizes the method of transforming the central node into a central edge set. This actually combines the advantages of the method of selecting seeds based on nodes and those of dividing communities based on edges. In the process of dividing non-central edges, the Jacard distance and the shortest distance between edges are combined to measure the distance between nonadjacent edges. Finally, the community is optimized, and a new pruning method is proposed for excessively overlapping nodes to make the division result more consistent for the real network. The NLC algorithm is applied to networks with real partitions and compared with classic algorithms and recent algorithms in terms of NMI, EQ and coverage rate (CR). The NLC algorithm gives slightly superior results compared to those of other algorithms. The results confirm that the algorithm can be used to find overlapping community structures in complex networks. Then, the algorithm was applied to the PPI networks to determine the overlapping community structures and perform functional enrichment analysis. The results of the enrichment analysis show that we can use the NLC algorithm to predict the function of the proteins in the PPI networks and find the overlapping structures in the protein functional modules.

## Methods

Complex networks are usually represented as graphs with nodes and edges. In a graph **G** = (**V**,**E**), **V** represents a set of nodes and **E** represents a set of edges.

### Community Detection Algorithm Based on Central Edge Selection (CES)

In 2019, Zhang et al. proposed the CES algorithm based on center-edge selection theory. It is necessary to briefly describe the basic idea of the CES algorithm, which consists of 3 steps: central edge selection, community division, and overlapping node pruning.

In the first step, the community magnetic interference theory (CMI) was used to improve the seed selection. In fact, this theory reduced the influence *F* of the neighboring nodes of the central node. The definition of the influence *F* was set as the following formula:


(1) $$F\left(v\right)=GF\times \sum_{u\in N\left(v\right)}IB\left(v,u\right)$$

(2) $$IB\left(n1,n2\right)=\frac{D\left(n1\right)\times D\left(n2\right)}{{\left(1-sim\left(n1,n2\right)\right)}^{2}}$$

where **N**(*v*) = {*u*|*u* ∈ **V**, (*v*, *u*) ∈ **E**}, *IB* (*n*1, *n*2) is the influence between the node *n*1 and the node *n*2, *GF* is the coefficient of CMI theory used to revise the value of *F*, *D*(*n*1) represents the degree of node *n*1, *D*(*n*2) is the degree of node *n*2, and $sim\left(n1,n2\right)=\frac{\left|N\left(n1\right)\cap N\left(n2\right)\right|}{\left|N\left(n1\right)\cup N\left(n2\right)\right|}$ represents the similarity between node *n*1 and node *n*2. The Formula (2) is derived from the universal gravitation formula $G=\frac{m_{1}\times m_{2}}{r^{2}}$.

The second step was to cluster non-central edges to the corresponding communities. This process was mainly divided according to the distance formula between edges. After the completion of edge division, the nodes were divided according to the edge division results.


(3) $$\begin{array}{c} DNC\left(e_{k},{\text{CE}}_{i}\right)=\\ \sum_{e_{j}\in {\mathrm{CE}}_{i}}\frac{ELC\left(e_{k},e_{j}\right)\times \left(\sum_{e_{m}\in {\mathrm{CE}}_{i}}ELC\left(e_{k},e_{m}\right)-ELC\left(e_{k},e_{j}\right)\right)}{\sum_{e_{m}\in {\mathrm{CE}}_{i}}ELC\left(e_{k},e_{m}\right)} \end{array}$$

where *DNC*(*e*_*k*_, **CE**_*i*_) represents the distance between edge *e*_*k*_ and central edge set **CE**_*i*_; *e*_*j*_ and *e*_*m*_ are the edges contained in the central edge set; and *ELC*(*e*_*k*_, *e*_*j*_) represents the similarity between edge *e*_*k*_ and edge *e_j_*, which is defined by the following formula.


(4) $$\begin{array}{c} ELC\left(e_{k},e_{j}\right)=ELC\left(e\left(a,b\right),e\left(c,d\right)\right)\\ =\left|\frac{N\left(a\right)\cap N\left(c\right)+N\left(a\right)\cap N\left(d\right)+N\left(b\right)\cap N\left(c\right)+N\left(b\right)\cap N\left(d\right)}{N\left(a\right)\cup N\left(c\right)+N\left(a\right)\cup N\left(d\right)+N\left(b\right)\cup N\left(c\right)+N\left(b\right)\cup N\left(d\right)}\right| \end{array}$$

The last step was pruning overlapping nodes. For all overlapping nodes, the proportion of non-central edges in the connection between the overlapping node and all communities was calculated and compared with a threshold. If the proportion was greater than the threshold, we could determine that the overlapping node belonged to the community.

### Limitation of CES

**FIGURE 1:**
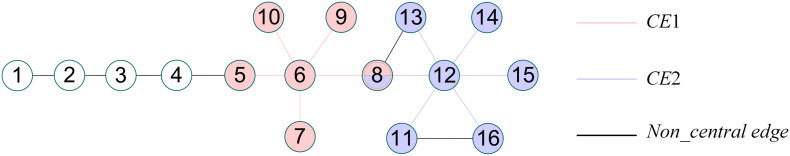
A simple network.

For the CES algorithm, some details need to be optimized. The selection of the seed node is not sufficiently accurate. In the process of clustering non-central edges, CES divides the non-central edges into the central edge set with the minimum distance. When measuring the distance between edges, the CES algorithm can only calculate the distance between edges where the topological distance is less than 3. For instance, in a small benchmark network containing 2 central edge sets and some non-central edges as Figure 1 shown. According to Formulas (3, 4), *DNC*(*e*(1, 2), **CE**_1_) = 0, *DNC*(*e*(1, 2), **CE**_2_) = 0. But *DNC*(*e*(1, 2), **CE**_1_) should be smaller than *DNC*(*e*(1, 2), **CE**_2_) because *e*(1, 2) is closer to **CE**_1_. And *e*(1, 2) should be divided into community 2. The CES algorithm cannot give a reasonable solution. In the pruning process of overlapping nodes, only the connection between the overlapping nodes and the central node are considered, but most nodes in the network are not connected with the central node. Further research is still important.

### Community Detection Algorithm Based on the Neighbor Local Clustering Coefficient (NLC)

**FIGURE 2:**
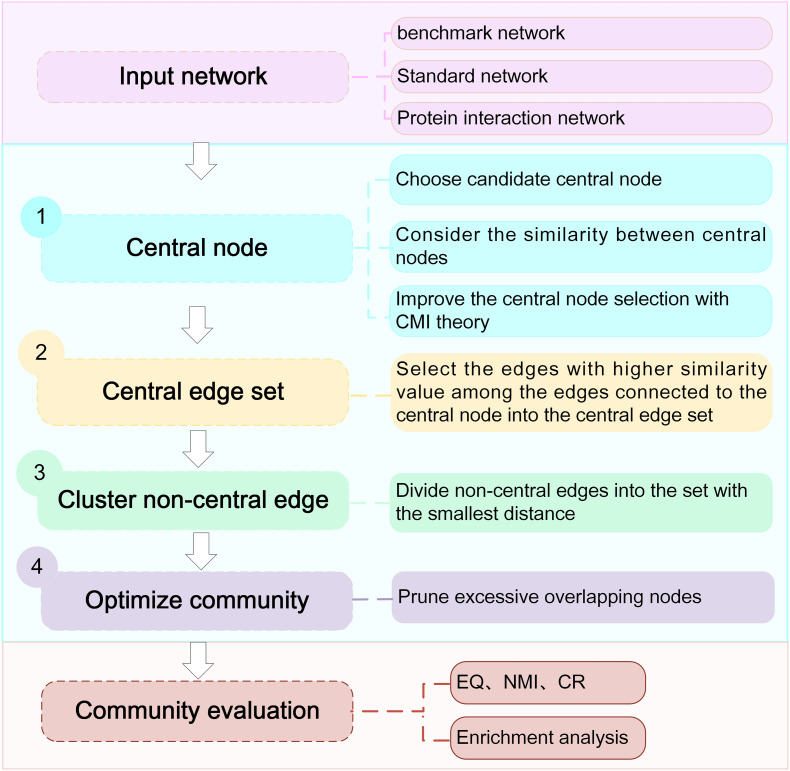
Workflow of the NLC algorithm.

To avoid the limitations of CES, we proposed the NLC algorithm. The algorithm combined the seed-based community detection algorithm with the edge-based community detection algorithm, which mainly consisted of four processes: (1) seed selection, (2) transformation from the central node set to the central edge set, (3) expansion of the non-central edge set, and (4) community optimization. The algorithm flow chart is as Figure 2 shown.

#### Central Node Selection

The selection of the central node could affect the result of community division. Inspired by the previous method of selecting central nodes, this paper introduced the neighbor local clustering coefficient and proposed a more reasonable selection method of central nodes. The process of selecting seeds is as follows:

1. First, the influence *F* of each node was calculated. If a node had a greater *F* than its neighboring nodes, then the node was considered as a candidate central node. The influence *F* of node *u* in the network is defined as the following formula.


(5) $$F\left(u\right)=\sum_{\begin{array}{c} v\in N\left(u\right)\\ v\neq u \end{array}}\frac{D\left(u\right)\times \left(1+C\left(u\right)\right)\times D\left(v\right)\times \left(1+C\left(v\right)\right)}{{\left(1-sim\left(u,v\right)\right)}^{2}}$$

where *C*(*u*) is the local clustering coefficient between the neighbors of node *u*. The local clustering coefficient quantified the clustering of neighboring nodes to form a cluster (complete graph). The clustering coefficient was defined as the following formula:


(6) $$\left\{ \begin{array}{c} C\left(u\right)=\frac{2K\left(u\right)}{\left|N\left(u\right)\right|\text{×}\left(\left|N\left(u\right)\right|-1\right)},\;\left|\text{N}\left(u\right)\right|\neq 1\\ C\left(u\right)=0,\left|\text{N}\left(u\right)\right|=1 \end{array}\right.$$

where *K*(*u*) represents the number of connections in the neighbor nodes. As shown in Figure 1, the calculation process of *C*(12) is as follows: **N**(12) = {8, 11, 13, 14, 15, 16}, the connected edges in the neighbors of node 12 are *e*(8, 13) and *e*(11, 16), so *K*(12) = 2, $C\left(12\right)=\frac{2\times 2}{6\times \left(6-1\right)}=0.13$.

2. A good community division results in close connections within the community and sparse connections between communities. Therefore, only when the similarity between the candidate central node and each central node is less than the threshold α, can the candidate central node be added to the central node set **CN**; that is, if *sim*(*n*1, *n*2) ≤ α *n*1 ∈ **CN**, then **CN** = **CN**∪{**n2**}, where *n*1 is a central node and *n*2 is a candidate central node.

3. The CMI theory in the CES algorithm was used to revise the weights of the neighbors of the central node, which reduced the possibility of the neighbors becoming the central node. We confirmed that the CMI theory could improve the selection of seed nodes.

#### The Transformation of the Central Node Set to the Central Edge Set

After selecting the central node, we chose edges that connect to central node and the similarity between two vertices was greater than the average similarity. As shown in Formulas (7, 8).


(7) CE={(u,v)|u∈CNandsim(u,v)>ave_sim(u)}

(8) $$\text{ave}\_sim\left(u\right)=\frac{1}{\left|\text{N}\left(u\right)\right|}\sum_{v\in N\left(u\right)}sim\left(u,v\right)$$

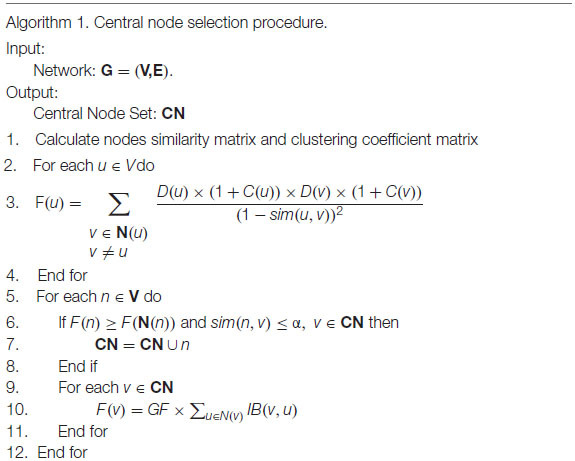


where *ave*_*sim* represents the average similarity between node *u* and its neighboring nodes, **CE** represents the central edge set, and **CN** represents the central node set. It can be concluded that each central node corresponds to a central edge set. The remaining edges are called non-central edges.

#### Clustering of Non-central Edge

After the central edge set was obtained, the remaining non-central edges could be clustered. The strategies of clustering non-central edges were as follows:

The distance between edges were calculated according to the Formula (9) and then the distance between the non-central edges and each central edge set was calculated. The non-central edge was clustered into the central edge set with the smallest average distance.


(9) Dis(a,b)=Jacard(a,b)×link(a,b)

(10) $$DLS\left(e,\mathrm{CE}\right)=\frac{\sum_{v\in \mathrm{CE}}Dis\left(e,v\right)}{\left|\mathrm{CE}\right|}$$

where *Jacard*(*a*, *b*) represents the *Jacard* distance of edge (*a*, *b*), *link*(*a*, *b*) is the topological distance of edge (*a*, *b*), and *DLS*(*e*,**CE**) represents the average distance between edge *e* and central edge set **CE**. To date, the community clustering of edges had been formed. Our next step was to transform the community division of edges into a community division of nodes. If multiple edges connected to a node belong to multiple communities simultaneously, then this node can be considered as an overlapping node, as shown in node 8 in Figure 1.

#### Community Optimization

There were a large number of overlapping nodes in the edge clustering results obtained in the previous process. Therefore, this paper proposed the following method to optimize these excessive overlapping nodes. We only needed to adjust the overlapping nodes in each community. So, the non-overlapping nodes were regarded as the divided parts, and the community was optimized by continuously reducing unnecessary overlapping nodes. The strategies were as follows, and the specific details were shown in algorithm 2.

The proportion of connection between the overlapping nodes and divided parts in each community was calculated. If the proportion was less than the pruning threshold *prune*, the overlapping node did not belong to the community; that is $\frac{con\left(n,\mathrm{Non}\_{\mathrm{overlap}}_{j}\right)}{\sum_{k\in clus\left(n\right)}con\left(n,\mathrm{Non}\_{\mathrm{overlap}}_{k}\right)}<prune$, where *n* ∉ *j*, **Non**_**overlap**_*j*_ represents the set of non-overlapping nodes in community *j*, *con*(*n*, **Non**_**overlap**_*j*_) represents the number of connections between overlapping node *n* and non-overlapping parts in the community *j*, and *clus*(*n*) represents the community to which overlapping node *n* belongs. If the connection proportion between the overlapping node and each community was less than the threshold *prune*, the overlapping node was only divided into the community with the largest connection proportion. If the size of the community was less than 3, the community was not pruned.


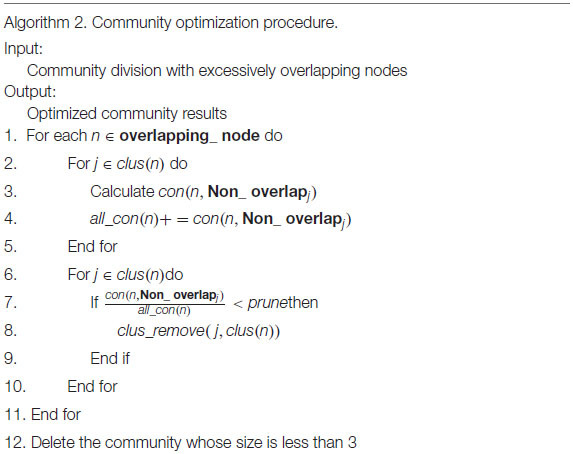


#### Time Complexity Analysis

Assuming that the network contains *n* nodes and *m* edges, in the power-law distribution, the degree of each node satisfies the distribution $P\left(degree=k\right)\propto \frac{1}{k^{\gamma }}$, where *k* represents the degree of the node. When the degree of a node is *k*, the probability of the node may be $\frac{1}{k^{\gamma }}$. In 2001, Béla Bollobás et al. proposed that the value of γ in large networks is generally always 3 (Bollobás et al., 2001). Therefore, the probability of existence of a node with degree *k* is $\frac{1}{k^{3}}$. The average degree in the network is $D\_ave=1\text{×}\frac{1}{1^{3}}+2\text{×}\frac{1}{2^{3}}+\cdots +n\text{×}\frac{1}{n^{3}}$. $\lim_{n\to \infty }\left(\frac{1}{1^{2}}+\frac{1}{2^{2}}+\cdots +\frac{1}{n^{2}}\right)=\frac{\pi^{2}}{6}$ (Dunham, 1999), so the total degree of all nodes is $DN=n\times D\_ave\leq \frac{\pi^{2}}{6}\times n$. In a network, the sum of the degrees of all vertices is equal to twice the number of edges in the graph, that is, m=$\frac{DN}{2}$≤$\frac{\pi^{2}}{12}$×n.

The first step of the NLC algorithm is to select the central nodes. First, we need to calculate the similarity between all nodes in the network, and the time complexity is *O*(*n*^2^). When choosing a central node, we need to access all nodes to calculate its *F*value and compare it with its neighboring nodes. The time is $O\left(\sum_{v\in V}\text{D}\left(v\right)\right)=\mathrm{DN}\leq \frac{\pi^{2}}{6}\times n$, where D(*v*) is the degree of node *v*. The second step of the algorithm is to transform the central node set into a central edge set, and the time is $O\left(\sum_{v\in \mathrm{CN}}E\left(v\right)\right)\leq \left|\mathrm{CN}\right|\text{×}\frac{\pi^{2}}{6}$, where **CN** is the central node set and *E*(*v*) is the size of the central edges of central node *v*. In the third step, the distance between edges in the network is calculated, and the time complexity is *O*(*m*^2^). The process of clustering non-central edges needs to calculate the distance between the non-central edges and each community, and the time complexity is *O*(*m*×|**CN**|). Finally, the process of community optimization requires calculating the proportion of non-overlapping parts of all the neighbors of overlapping nodes in each community, and the time requires ∑_*v* ∈ **overlapping**_**node**_ *D*(*v*). Through the above analysis, after omitting the constant of the highest order position, the time complexity of the NLC algorithm is 0(*n*^2^).

## Results and Discussion

### Datasets

(1) Standard networks

The standard networks used in this paper were Zachary’s karate club (Zachary, 1977), American college football (Girvan and Newman, 2002), and books about US politics (polbooks) (Tang, 2014), which are all networks with standard divisions. The karate network is a social network of friendships between 34 members of a karate club at a US university in the 1970s. Each node represents a student, each edge represents the communication relationship between students, and each community represents a team led by a coach. The football network is a network of American football games between Division IA colleges during the regular Fall 2000 season. Each node represents a player, an edge represents a match between players, and a category represents a collection of teams. The polbooks network is a network of books about US politics published around the time of the 2004 presidential election and sold by the online bookseller Amazon.com. Edges between books represent frequent co-purchasing of books by the same buyers. The specific conditions of each network are shown in Table 1, where NSC represents the number of standard communities.

**TABLE 1:** Standard networks.

Standard networks	|E|	|V|	NSC
Karate	78	34	2
Football	612	115	12
Polbooks	105	441	3

(2) Benchmark networks

**FIGURE 3:**
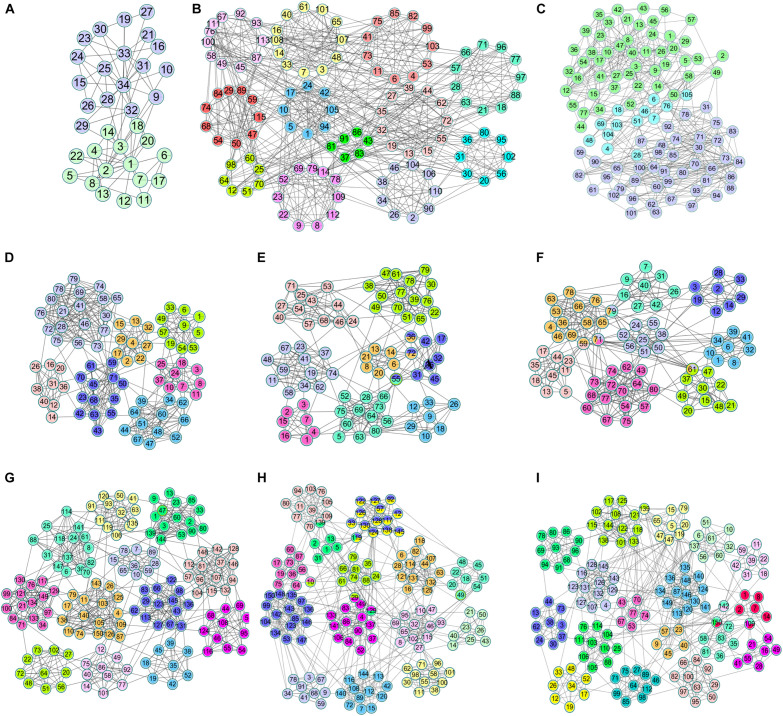
The visualization of standard networks and LFR networks. Standard networks **(A)** Karate network, **(B)** Football network, **(C)** Polbooks network; LFR networks: **(D)** LFR1 network, **(E)** LFR2 network, **(F)** LFR3 network, **(G)** LFR4 network, **(H)** LFR5 network, **(I)** LFR6 network.

Compared with real world networks, artificial synthetic networks can more effectively measure the accuracy of detected community divisions because they can predict the real network micro characteristics and community divisions (Ren et al., 2019). This paper used the LRF benchmark network to synthesize the network, which was a benchmark method for testing the performance of the algorithm found in the community (Lancichinetti et al., 2008). LFR networks have multiple parameters to control the structure and scale of the synthesized network. The commonly used parameters in LFR are N (number of nodes), K (average degree, the average degree of most large-scale real social networks is approximately 10), Maxk (maximum degree), Mu (mixing parameter), On (number of overlapping nodes), and Om (number of memberships of the overlapping nodes). In this paper, there were 6 networks used for experiments, including two types of networks with different numbers of nodes and different overlapping ratios. The specific parameters and the generated network information were shown in Table 2. The visualization results of standard networks and LFR networks were shown in Figure 3. The visualization of the network in this paper was drawn by Cytoscape (Shannon et al., 2003). In the visualized results, different colors represented different communities.

**TABLE 2:** LFR benchmark networks.

LFR benchmark networks	|V|	Maxk	Mu	K	On	Om	|E|	NSC
LFR1	80	15	0.1	10	4	1	764	7
LFR2	80	15	0.1	10	4	2	740	8
LFR3	80	15	0.1	10	4	3	778	8
LFR4	150	15	0.1	10	8	1	1,418	12
LFR5	150	15	0.1	10	8	2	1,478	14
LFR6	150	15	0.1	10	8	3	1,426	17

(3) PPI networks

The PPI networks used in the experiment were all downloaded from the database of interacting proteins (DIP) (Salwinski et al., 2004). The reliability of the DIP database is high, because it only stores protein interaction verified by experiments, and provides experimental methods used to identify the interaction. The DIP lists protein pairs that are known to interact with each other and are composed of nodes and edges. The nodes represent proteins, and the edges represent the interactions between proteins. The downloaded network version was 20170205 and the downloaded networks were: *M. musculus*, *H. sapiens*, *D. melanogaster*, and *R. norvegicus*. These networks are real networks without standard communities. The unprocessed PPI networks contain some redundant edges and some small structures, so this noise needed to be processed in data processing: the self-circulating edges in the network and some modules with a small scale were removed. The number of nodes and edges of the PPI networks before and after data preprocessing are shown in Table 3.

**TABLE 3:** The PPI networks.

	Before data preprocessing		After data preprocessing	
PPI networks	|E|	|V|	|E|	|V|
*H. sapiens*	7,380	4,670	6,699	4,200
*M. musculus*	2,597	2,329	2,319	2,006
*D. melanogaster*	711	626	614	518
*R. norvegicus*	619	665	497	504

### Evaluation Metrics

To verify whether the community structure detected by the algorithm was reasonable, the algorithm was compared with the CES algorithm, CNS algorithm, CPM algorithm and LC algorithm. The CES algorithm was an edge partition-based algorithm proposed in 2019, and CNS was an algorithm based on node division proposed in 2017. The CPM algorithm and LC algorithm were relatively classic algorithms in the field of overlapping community discovery. The software CFinder (version 2.0.6) is a free software for finding and visualizing overlapping communities, based on the CPM. The clustering result of LC algorithm was obtained by the linkcommon package which includes tools for generating, visualizing, and analyzing overlapping communities (Kalinka and Tomancak, 2011). These algorithms were compared and analyzed with the standard networks, LFR synthesis networks and PPI networks to evaluate the accuracy of this algorithm. To evaluate the performance of the NLC algorithm, we used the following 5 evaluation indicators.

(1) Extended modularity (EQ)

Since the community structure of the complex network was unknown in advance, a metric was needed to measure the community results detected by different community detection algorithms. In this paper, the extended modularity (EQ) (Shen et al., 2009) evaluated the results of overlapping community detections. The value of EQ can be calculated by Formula (11):


(11) $$EQ=\frac{1}{2\left|E\right|}\sum_{i=1}^{\left|C\right|}\sum_{v,w\in C}\frac{1}{OvOw}\left(A_{vw}-\frac{{\text{D}}_{v}{\text{D}}_{w}}{2\left|E\right|}\right)$$

where |**C**| represents the number of communities detected, *Ov* represents the number of communities to which node *v* belongs, *Ow* represents the number of communities to which node *w* belongs, and *Avw* varies according to different situations: when the node *v* is connected to the node *w*, *Avw* = 1; otherwise, *Avw* = 0. The EQ value is between 0 and 1, and a larger value is better.

(2) Normalized Mutual Information (NMI)

The normalized mutual information (NMI) used in this paper is proposed by Lancichinetti et al. (2009) and widely used in overlapping community evaluations, which is defined as the following formula:


(12) NMI(R|P)=1-[H(R|P)+H(P|R)]/2

where *R* is the real community, *P* is the predicted division result, and *H*(*R*|*P*) is the normalized conditional entropy of *R* with respect to *P*. The NMI value is between 0 and 1; the closer the value is to 1, the closer it is to the real community. The NMI value is 1 when the result of community division matches the real community completely.

(3) Coverage Rate (CR)

The coverage rate is used to evaluate the coverage of community detection, which is defined as the following formula.


(13) $$CR=\frac{n^{\prime }}{n}\cdot 100\%$$

where *n*′ represents the number of nodes detected by the community detection algorithm and *n* represents the total number of nodes in the network.

(4) Number of Normalized Communities (NNC)

The NNC is used to evaluate the difference between the true and predicted values, which is defined as Formula (14):


(14) $$NNC=\text{max}\left(1-\frac{\left|NSC-NPC\right|}{NSC},0\right)$$

where NSC represents the number of standard communities and NPC represents the number of communities predicted by algorithms. The NNC value is between 0 and 1; the closer the value is to 1, the closer it is to the number of standard communities. When the NNC value is 1, the predicted number of communities is consistent with the actual number of communities.

(5) Enrichment analysis

To detect whether the community detected by the algorithm has biological significance, functional enrichment analysis of the protein community is necessary. Enrichment analysis of a gene set refers to comparing the gene set to a database that is classified and annotated according to prior knowledge, using the hypergeometric distribution algorithm to obtain the gene ontology terms with significant enrichment of genes of the gene set. The gene ontology term corresponding to the smallest *p*-value was used as the functional annotation of the protein community. Among these databases, Gene Ontology (GO) (Ashburner et al., 2000) and Kyoto Encyclopedia of Genes and Genomes (KEGG) (Kanehisa and Goto, 2000) are commonly used. The GO annotation contains three indicators: biological process (BP), cellular component (CC) and molecular function (MF). BP describes the biological processes in which proteins are involved. CC describes the location of the proteins in the cell for biological activity. MF describes the biochemical activity of proteins. KEGG provides a complete metabolic pathway, including the metabolism of carbohydrates, nucleosides, and amino acids. and the biodegradation of organic matter. The values of the above four indicators are all expressed by *p*-values, where the closer the *p*-value is to 0, the more significant the biological significance of the divided communities is. During the experiment, the cluster profiler of the R package was used for enrichment analysis (Yu et al., 2012).

### Experimental Setup

#### Parameters in the NLC Algorithm

**FIGURE 4:**
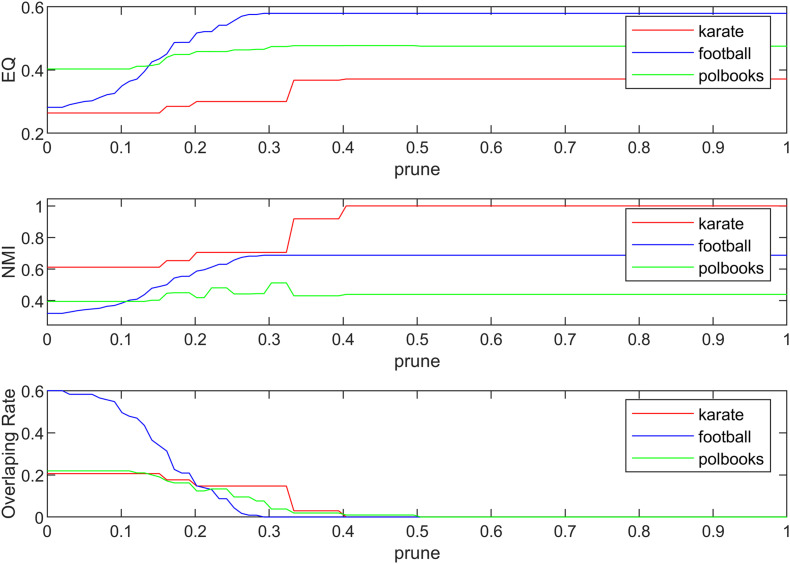
The relationship between EQ, NMI, OR and *prune* in the standard networks.

**FIGURE 5:**
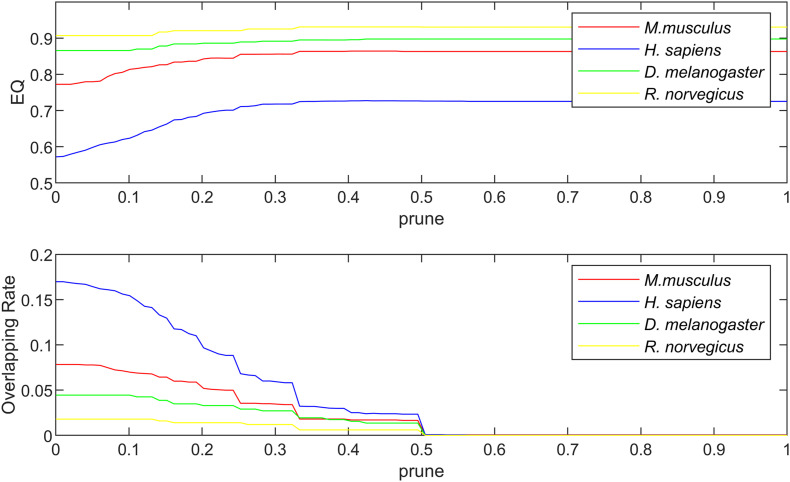
The relationship between EQ, OR and *prune* in the PPI networks.

The algorithm proposed in this paper mainly involves three parameters, which are the community magnetic interference coefficient *GF*, similarity threshold α and pruning coefficient *prune*. The similarity threshold α prevented excessive similarity between two communities. The similarity threshold α was tested between 0 and 1. According to experimental experience, in the karate network α = 0.14; in the football network α = 0.30; and in the polbooks network α = 0.10; the threshold α values of four PPI networks were set to 0.1. The parameter *GF*was used to control the centrality of the neighboring nodes of the central node and was set as $GF=\frac{link\_num}{node\_num}$. The pruning coefficient *prune* reduced the excessive overlapping nodes, and the value was between 0 and 1 for the experiment. The relationship between coefficient *prune*, EQ, NMI and overlapping rate (OR) in the standard networks and PPI networks were shown in Figures 4, 5. The OR was used to describe the proportion of overlapping nodes in the community.

As is demonstrated in Figure 4, we can see that different values of parameter *prune* can have various influences on the experiment result. The selection of *prune* was based on the EQ, NMI and *OR*-values. If there is no overlapping structure in the network, we only need to select the corresponding parameters when the EQ and NMI values are relatively good; if there is an overlapping structure in the network, we also need to consider the overlapping structure in the network. Finally, in the three networks of karate, football, and polbooks, *prune* were selected as 0.42, 0.30, 0.31, respectively.

Figure 5 shows the relationship between EQ, OR and *prune* in the PPI networks. The selection of *prune* was based on the value of EQ and OR. In the PPI networks, some proteins have multiple functions and form protein overlapping nodes. Hence, we need to maintain some overlapping structures while the value of EQ is high. In the four PPI networks, the *prune* values were set as 0.32.

#### The Experimental Results on Networks With Standard Division

**FIGURE 6:**
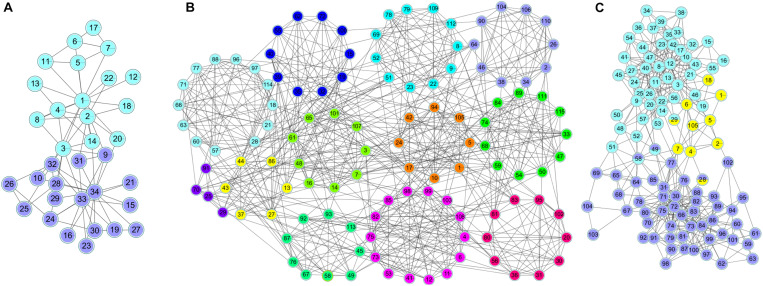
The division results of the NLC algorithm in three standard networks: **(A)** Karate, **(B)** Football, and **(C)** Polbooks network.

Figure 6 shows the clustering results of the karate, football and polbooks networks based on the NLC algorithm. Colors represent communities, and nodes with overlapping colors represent that they can belong to multiple communities.

**FIGURE 7:**
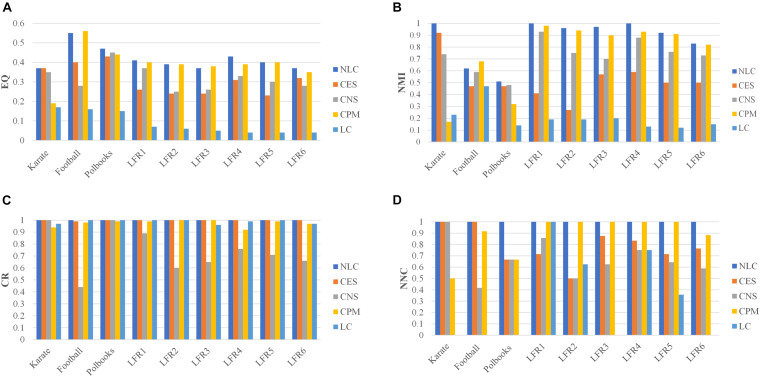
The division results of five algorithms in standard networks and LFR networks including three indicators: **(A)** EQ, **(B)** NMI, **(C)** CR, and **(D)** NNC.

The NLC algorithm was compared with other four algorithms including CES, CNS, CPM, and LC, by comparing the EQ, NMI, CR and NNC values in networks with standard division: the karate, football, polbooks, and LFR networks. The results were shown in Figure 7.

In the CPM algorithm, we set the value of *k* as 3 in the karate, football and polbooks networks; we set the *k* as 5 in the LFR networks. In the CES algorithm, we set the coefficient $GF=4.2\text{×}\frac{link\_num}{node\_num}$ in three standard networks. The parameter in the CNS algorithm was set to 0.4 according to Qi (Qi et al., 2017). In the karate, football, polbooks, LFR 3 and LFR 6 networks, the number of predicted communities (NPC) by the LC algorithm were quite different from the actual number of communities, so the NNC values were 0 in these networks. And the values of NPC obtained by five algorithms were shown in the Supplementary Materials. The NLC algorithm could completely pair the karate network and had the best EQ value and NMI value. In the football network, the CPM algorithm had the best *EQ*-value and *NMI*-value but the *NNC*-value was smaller than the NLC. In the polbooks network, the NLC algorithm also had the best EQ and NMI. The NLC algorithm could completely pair LFR synthetic networks (LFR1 and LFR4) without overlapping nodes. In the LFR3 network, the CPM algorithm had the highest *EQ*-value, but the NLC algorithm had the highest *NMI*-value. In the LC algorithm, The NLC algorithm not only had good division results in the LFR network with overlapping structures but also could be applied in the non-overlapping networks. In general, the NLC algorithm had better division result than the other four algorithms.

#### The Experimental Results of PPI Networks

**FIGURE 8:**
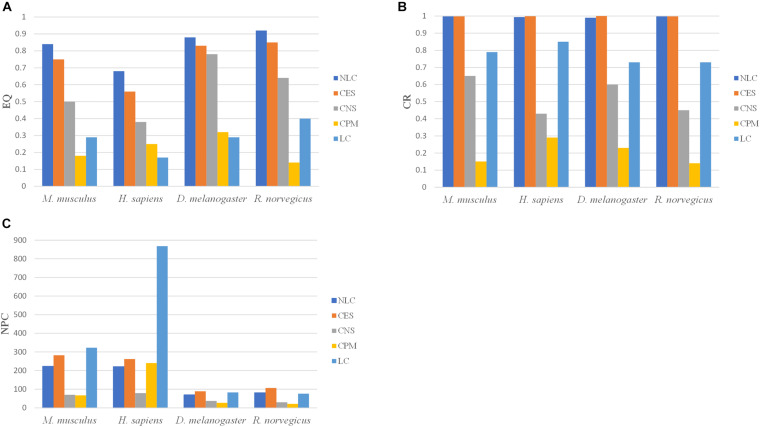
The division results of five algorithms in PPI networks including three indicators: **(A)** EQ, **(B)** CR, and **(C)** NPC.

The calculation of NMI and NNC requires not only the predicted communities, but also the real communities. Since the real communities in the PPI networks is unknown, the NMI and NNC metrics cannot be calculated. The NLC algorithm was compared with the CES, CNS, CPM, and LC algorithms, by comparing the EQ, CR and NPC values in the four PPI networks: *M. musculus, H. sapiens, D. melanogaster* and *R. norvegicus.* The results were shown in Figure 8.

In the CPM algorithm, the parameter *k* was set to 3 in four PPI networks. In the CES algorithm, the parameter *GF* in *M. musculus*, *H. sapiens*, *D. melanogaster*, and *R. norvegicus* networks were set to 0.8, 0.3, 0.3, 0.8, respectively. The clustering results of the NLC had higher EQ and CR values in the four PPI networks than the other algorithms. The CPM and LC algorithms had the smallest EQ. The division categories of the algorithm proposed in this paper were always at an intermediate value when compared with other algorithms, indicating that the divided community structure obtained by this algorithm was relatively more reasonable. Moreover, the division effect of the developed algorithm was better than that of the other four algorithms from the perspective of EQ and CR values.

**FIGURE 9:**
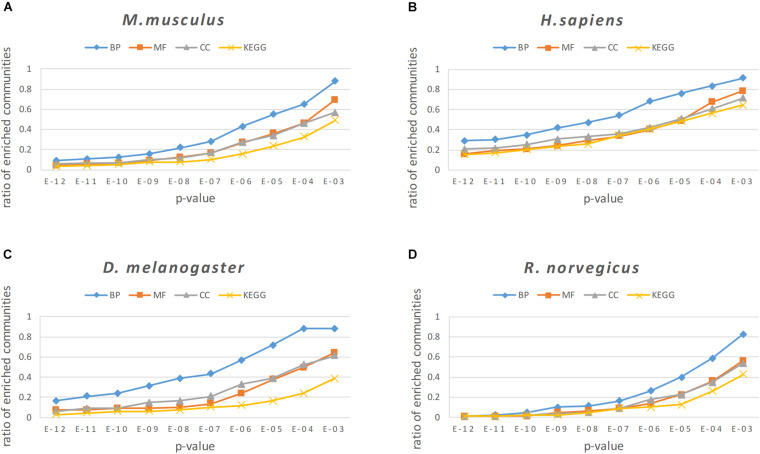
The enrichment analysis results of the NLC in four PPI networks: **(A)** *M. musculus*, **(B)** *H. sapiens*, **(C)** *D. melanogaster*, and **(D)** *R. norvegicus.*

For enrichment analysis, it was necessary to calculate the *p*-value of the BP, MF, CC categories and KEGG pathways for each protein community, and the smallest *p*-value is selected as the result of enrichment analysis for a particular protein community. To better reflect the enrichment result of the protein community, communities with more than 2 proteins were left, because the communities with only two proteins are more likely to generate noise on the enrichment results. In our experiment, we set the threshold of the *p*-value as 0.05. Generally, the gene or protein was considered to be significantly expressed when the *p* < 0.05; otherwise, the community was regarded as an insignificant expression community. In Figure 9, a *p*-value threshold sequence of 1E-12 to 1E-03 was set, and the proportion of modules less than or equal to this *p*-value threshold was counted for all protein modules found in the four PPI networks.

From the results of enrichment analysis shown in Figure 9, the algorithm proposed in this paper obtains good enrichment results in the BP, MF, and CC classes and KEGG pathways. The BP analytical result was the best in the enrichment analysis, indicating that proteins in the protein community identified by the algorithm in this paper had a high degree of co-participation in biological processes. The BP analytical results show that 97.6% of the communities in the *M. musculus* network had a p-value ≤ 1*E* − 02, 87.4% communities had a p-value ≤ 1*E* − 03, and the proportion of communities with a p-value ≤ 1*E* − 02 in the three networks of *H. sapiens*, *D. melanogaster*, and *R. norvegicus* were 91.4, 88.1, 82.5%, respectively.

**FIGURE 10:**
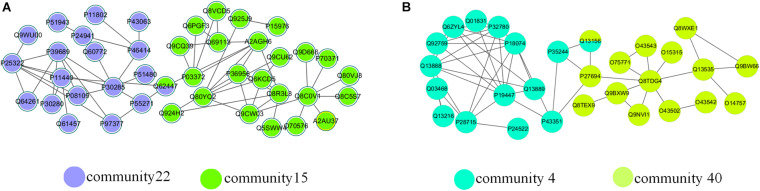
Overlapping structures in **(A)** *M. musculus* network and **(B)** *H. sapiens* network.

There were a large number of overlapping communities in the division results of the NLC algorithm. Taking the *M. musculus* and the *H. sapiens* networks as examples, Table 5 and Supplementary Table 1 list the enrichment analysis results of some overlapping communities divided by the NLC algorithm, including the GO ID enriched in the protein community and its functional description, which is the definition of GO terms. Figure 10 depicts the visual results.

In Table 4, the ID is the unique identifier for the GO database or KEGG database. There was an overlapping node Q62447 between communities 15 and 22 divided by the NLC algorithm, and the corresponding protein name is Cyclin-C. Cyclin-C is a component of the mediator complex, which is a coactivator involved in the regulation of gene transcription of almost all RNA polymerase II-dependent genes. Its molecular function is related to the cyclin-dependent protein serine, and there are four biological processes related to Cyclin-C: negative regulation of triglyceride metabolism, positive regulation of RNA polymerase II transcription, protein ubiquitin chemical and RNA polymerase II regulates transcription (Gaudet et al., 2011). Through enrichment analysis, we found that the molecular function of community 15 was the activity of ubiquitin protein ligase, and the cell composition was related to the composition of the mediator complex. The protein Cyclin-C also had the function of community 15. The cellular component of community 22 was a cyclin-dependent protein kinase holoenzyme compound. Its molecular function was to regulate the activity of cyclin-dependent protein serine/threonine kinase. The protein Cyclin-C also had the molecular function of community 22.

**TABLE 4:** Enrichment analysis of an overlapping structure in the *M. musculus* network.

Overlapping communities in the *M. musculus* network	Enrichment analysis	*p*-value	ID	Name
Community 15	BP	1.14E-14	GO:0098813	Nuclear chromosome segregation
	MF	2.26E-09	GO:0061630	Ubiquitin protein ligase activity
	CC	5.59E-18	GO:0016592	Mediator complex.
	KEGG	2.98E-06	mmu04114	Oocyte meiosis
Community 22	BP	8.80e-19	GO:0044843	Cell cycle G1/S phase transition.
	MF	9.69e-24	GO:0016538	Cyclin-dependent protein serine.
	CC	6.31e-21	GO:0000307	Cyclin-dependent protein kinase holoenzyme complex.
	KEGG	1.13e-22	mmu04110	Cell cycle.

As we can see from the Table 5, the biological function of community 4 is DNA binding, and the biological function of community 40 is the excision of nucleotides. The overlapping node between community 4 and community 40 is Q13156, which corresponds to RPA4. The biological functions of RPA4 are participation in single-stranded DNA binding, DNA replication and repair, double-strand break repair via homology, DNA damage checkpoint (Haring et al., 2010), DNA replication initiation (Keshav et al., 1995), Nucleotide excision repair (Kemp et al., 2010). The overlapping protein Q13156 has both the biological function of communities 4 and 40.

**TABLE 5:** Enrichment analysis of an overlapping structure in the *H. sapiens* network.

Overlapping communities in the *H. sapiens* network	Enrichment analysis	*p*-value	ID	Name
Community 4	BP	4.23e-16	GO:0000724	Double-strand break repair via homologous recombination
	MF	1.67e-12	GO:0000400	Four-way junction DNA binding.
	CC	4.18e-15	GO:0033061	DNA recombinase mediator complex.
	KEGG	1.37e-13	hsa03440	Homologous recombination
Community 40	BP	3.38e-31	GO:0006289	Nucleotide-excision repair
	MF	7.17e-17	GO:0008353	RNA polymerase II CTD heptapeptide repeat kinase activity
	CC	2.20e-22	GO:0005675	Transcription factor TFIIH holo complex.
	KEGG	9.96e-31	hsa03420	Nucleotide excision repair

By analyzing the two examples of overlapping communities in the *M. musculus* and *H. sapiens* networks above, we can conclude that the biological function of the overlapping protein is related to the biological function of the community where it is located, so we can use the algorithm proposed to predict the functions of overlapping proteins.

## Conclusion

This paper proposes an overlapping community detection algorithm based on the neighbor clustering coefficient to select the central edge. First, the node with the largest local influence in the network was found and determined as the central node. The central node was converted into central edge set. Then, the non-central edge was assigned to the community with the smallest distance. Finally, the community was optimized, and the excessively overlapping nodes were pruned according to the pruning strategy. The experimental results of the five algorithms on three types of networks show that the EQ and CR values of the NLC algorithm in this paper were improved and could identify overlapping structures better than the previously established algorithms. Applying the NLC algorithm to PPI networks can help us find overlapping structures in protein functional modules and discover unknown functions of proteins. In future work, we will continue to improve the algorithm so that it can adapt to changes in dynamic networks and further explore the application of the algorithm in biological information.

## Data Availability Statement

The original contributions presented in the study are included in the article/Supplementary Material, further inquiries can be directed to the corresponding author/s.

## Author Contributions

QC collected the data and performed the experiments. YW conceived the project and designed the study. LY, KH, SY, and XX wrote the manuscript. All authors read and approved the final manuscript for publication.

## Conflict of Interest

The authors declare that the research was conducted in the absence of any commercial or financial relationships that could be construed as a potential conflict of interest.

## References

[B1] Ahn Y.-Y. Bagrow J. P. Lehmann S. (2010). Link communities reveal multiscale complexity in networks. *Nature* 466 761–764. 10.1038/nature09182 20562860

[B2] Ashburner M. Ball C. A. Blake J. A. Botstein D. Butler H. Cherry J. M. (2000). Gene ontology: tool for the unification of biology. *Nat. Genet.* 25 25–29. 10.1038/75556 10802651 PMC3037419

[B3] Bhowmick S. S. Seah B. S. (2015). Clustering and summarizing protein-protein interaction networks: a survey. *IEEE Trans. Knowl. Data Eng.* 28 638–658. 10.1109/tkde.2015.2492559

[B4] Bollobás B. e Riordan O. Spencer J. Tusnády G. (2001). The degree sequence of a scale−free random graph process. *Rand. Struct. Algorith.* 18 279–290. 10.1002/rsa.1009

[B5] Chen Y. Wang W. Liu J. Feng J. Gong X. (2020). Protein interface complementarity and gene duplication improve link prediction of protein-protein interaction network. *Front. Genet.* 11:291. 10.3389/fgene.2020.00291 32300358 PMC7142252

[B6] Dunham W. (1999). *Euler the Master of Us All.* Washingdon, DC: Mathematical Association of America 15:28.

[B7] Gaudet P. Livstone M. S. Lewis S. E. Thomas P. D. (2011). Phylogenetic-based propagation of functional annotations within the Gene Ontology consortium. *Briefi. Bioinform.* 12 449–462. 10.1093/bib/bbr042 21873635 PMC3178059

[B8] Girvan M. Newman M. E. J. (2002). Community structure in social and biological networks. *Proc. Natl. Acad. Sci. U.S.A.* 99 7821–7826. 10.1073/pnas.122653799 12060727 PMC122977

[B9] Gu L. Han Y. Wang C. Chen W. Jiao J. Yuan X. (2019). Module overlapping structure detection in PPI using an improved link similarity-based Markov clustering algorithm. *Neural Comput. Appl.* 31 1481–1490. 10.1007/s00521-018-3508-z

[B10] Haring S. J. Humphreys T. D. Wold M. S. (2010). A naturally occurring human RPA subunit homolog does not support DNA replication or cell-cycle progression. *Nucleic Acids Res.* 38 846–858. 10.1093/nar/gkp1062 19942684 PMC2817474

[B11] Huang L. Li Y. Wang G. S. Wang Y. (2016). Community detection method based on vertex distance and clustering of density peaks. *J. Jilin Univ. Eng. Technol. Edn.* 46 2042–2051.

[B12] Ji J. Zhang A. Liu C. Quan X. Liu Z. (2012). Survey: functional module detection from protein-protein interaction networks. *IEEE Trans. Knowl. Data Eng.* 26 261–277. 10.1109/tkde.2012.225

[B13] Kalinka A. T. Tomancak P. (2011). linkcomm: an R package for the generation, visualization, and analysis of link communities in networks of arbitrary size and type. *Bioinformatics* 27 2011–2012. 10.1093/bioinformatics/btr311 21596792 PMC3129527

[B14] Kanehisa M. Goto S. (2000). KEGG: kyoto encyclopedia of genes and genomes. *Nucleic Acids Res.* 28 27–30. 10.1093/nar/28.1.27 10592173 PMC102409

[B15] Kemp M. G. Mason A. C. Carreira A. Reardon J. T. Haring S. J. Borgstahl G. E. (2010). An alternative form of replication protein a expressed in normal human tissues supports DNA repair. *J. Biol. Chem.* 285 4788–4797. 10.1074/jbc.M109.079418 19996105 PMC2836084

[B16] Keshav K. F. Chen C. Dutta A. (1995). Rpa4, a homolog of the 34-kilodalton subunit of the replication protein A complex. *Mol. Cell Biol.* 15 3119–3128. 10.1128/MCB.15.6.3119 7760808 PMC230543

[B17] Lancichinetti A. Fortunato S. Kertész J. (2009). Detecting the overlapping and hierarchical community structure in complex networks. *N. J. Phys.* 11:033015. 10.1088/1367-2630/11/3/033015

[B18] Lancichinetti A. Fortunato S. Radicchi F. (2008). Benchmark graphs for testing community detection algorithms. *Phys. Rev.* 78 046110. 10.1103/PhysRevE.78.046110 18999496

[B19] Mao Y. Liu Y. (2020). Functional module mining in uncertain PPI network based on fuzzy spectral clustering. *J. Comput.* 31 91–106. 10.3966/199115992020083104008

[B20] Palla G. Derényi I. Farkas I. Vicsek T. (2005). Uncovering the overlapping community structure of complex networks in nature and society. *Nature* 435 814–818. 10.1038/nature03607 15944704

[B21] Qi J. Xun L. Yi W. Information S. O. (2017). Overlapping community detection algorithm based on selection of seed nodes. *Appl. Res. Comput.* 34 3534–3537. 10.1016/j.compeleceng.2018.03.012

[B22] Ren H. Xiao J. Cui W. Xu X. (2019). Construction and applications of benchmark networks for community detection based on null models. *J. Univ. Electr. Sci. Technol. China* 48 440–448.

[B23] Salwinski L. Miller C. S. Smith A. J. Pettit F. K. Bowie J. U. Eisenberg D. (2004). The database of interacting proteins: 2004 update. *Nucleic Acids Res.* 32(suppl 1) D449–D451. 10.1093/nar/gkh086 14681454 PMC308820

[B24] Shannon P. Markiel A. Ozier O. Baliga N. S. Wang J. T. Ramage D. (2003). Cytoscape: a software environment for integrated models of biomolecular interaction networks. *Genome Res.* 13 2498–2504. 10.1101/gr.1239303 14597658 PMC403769

[B25] Shen H. Cheng X. Cai K. Hu M.-B. (2009). Detect overlapping and hierarchical community structure in networks. *Phys. A Stat. Mech. Appl.* 388 1706–1712. 10.1016/j.physa.2008.12.021

[B26] Tang X. (2014). *A Network of Books About US Politics Published Around the Time of the 2004.* New York, NY: Springer international publishing. 10.6084/m9.figshare.1149952.v1

[B27] Vella D. Marini S. Vitali F. Di Silvestre D. Mauri G. Bellazzi R. (2018). MTGO: PPI network analysis via topological and functional module identification. *Sci. Rep.* 8:5499. 10.1038/s41598-018-23672-0 29615773 PMC5882952

[B28] Ying K. Lin S. (2020). Maximizing cohesion and separation for detecting protein functional modules in protein-protein interaction networks. *PLoS One* 15:e0240628. 10.1371/journal.pone.0240628 33048996 PMC7553341

[B29] Yu G. Wang L. Han Y. He Q. (2012). clusterProfiler: an R package for comparing biological themes among gene clusters. *Omics* A *J. Integr. Biol.* 16 284–287. 10.1089/omi.2011.0118 22455463 PMC3339379

[B30] Zachary W. W. (1977). An information flow model for conflict and fission in small groups. *J. Anthropol. Res.* 33 452–473.

[B31] Zhang F. Ma A. Wang Z. Ma Q. Liu B. Huang L. (2018). A central edge selection based overlapping community detection algorithm for the detection of overlapping structures in protein–protein interaction networks. *Molecules* 23 2633. 10.3390/molecules23102633 30322177 PMC6222769

