# Corrigendum: Overlapping Structures Detection in Protein-Protein Interaction Networks Using Community Detection Algorithm Based on Neighbor Clustering Coefficient

**DOI:** 10.3389/fgene.2021.799818

**Published:** 2021-11-26

**Authors:** Yan Wang, Qiong Chen, Lili Yang, Sen Yang, Kai He, Xuping Xie

**Affiliations:** ^1^ Key Laboratory of Symbol Computation and Knowledge Engineering of Ministry of Education, College of Computer Science and Technology, Jilin University, Changchun, China; ^2^ School of Artificial Intelligence, Jilin University, Changchun, China; ^3^ Department of Obstetrics, The First Hospital of Jilin University, Changchun, China

**Keywords:** protein-protein interaction network, overlapping structure, community detection, central edge, clustering coefficient

In the original article, an author name was incorrectly written as “Chen Qiong”. It has been corrected to “Qiong Chen”.

Also, there was a mistake in “Funding” section as published. Funding grant numbers for “Development Project of Jilin Province of China” have been changed to “Nos. 20200401083GX, 2020C003,20200403172SF and another funder has been added: “Chinese Postdoctoral Science Foundation (No.801212011421).” The corrected statement appears below: This work was supported by the National Natural Science Foundation of China (Nos. 62072212 and 61902144), the Development Project of Jilin Province of China (Nos. 20200401083GX, 2020C003,20200403172SF) and Chinese Postdoctoral Science Foundation (No.801212011421).

The authors apologize for this error and state that this does not change the scientific conclusions of the article in any way. The original article has been updated.

