# Supplementary material for: Overlapping Structures Detection in Protein-Protein Interaction Networks Using Community Detection Algorithm Based on Neighbor Clustering Coefficient
Source: Front Genet. 2021 Jun 23;12:689515. doi: 10.3389/fgene.2021.689515 (PMC8261288; doi:10.3389/fgene.2021.689515)
Supplement: Supplementary file 1 [file Table_1.docx]

Supplementary Material

Table 1. The NPC obtained by five algorithms

| Networks | NLC | CES | CNS | CPM | LC |
| --- | --- | --- | --- | --- | --- |
| Karate (NSC=2) | **2** | **2** | **2** | 3 | 13 |
| Football (NSC=12) | **12** | **12** | 5 | 13 | 46 |
| Polbooks (NSC=3) | **3** | 2 | 2 | 4 | 34 |
| LFR1(NSC=7) | **7** | 9 | 6 | **7** | **7** |
| LFR2(NSC=8) | **8** | 4 | 4 | **8** | 11 |
| LFR3(NSC=8) | **8** | 9 | 5 | **8** | 17 |
| LFR4(NSC=12) | **12** | 14 | 9 | **12** | 15 |
| LFR5(NSC=14) | **14** | 10 | 9 | **14** | 23 |
| LFR6(NSC=17) | **17** | 13 | 10 | 15 | 32 |
| *M. musculus* | 223 | 282 | 70 | 67 | 323 |
| *H. sapiens* | 221 | 262 | 79 | 240 | 868 |
| *D. melanogaster* | 70 | 89 | 37 | 27 | 83 |
| *R. norvegicus* | 81 | 107 | 30 | 21 | 76 |
